# Improving quality of care in general practices by self-audit, benchmarking and quality circles

**DOI:** 10.1007/s00508-016-1064-z

**Published:** 2016-09-06

**Authors:** Angelika Mahlknecht, Muna E. Abuzahra, Giuliano Piccoliori, Nina Enthaler, Adolf Engl, Andreas Sönnichsen

**Affiliations:** 1 Institut für Allgemein-, Familien- und Präventivmedizin, Paracelsus Medizinische Universität Salzburg, Strubergasse 21, 5020 Salzburg, Austria; 2 Institut für Allgemeinmedizin und evidenzbasierte Versorgungsforschung, Medizinische Universität Graz, Auenbruggerplatz 2/9, 8036 Graz, Austria; 3 Südtiroler Akademie für Allgemeinmedizin, Wangergasse 18, 39100 Bolzano, Italy; 4 Institut für Allgemeinmedizin und Familienmedizin, Universität Witten/Herdecke, Alfred-Herrhausen-Str. 50, 58448 Witten, Germany

**Keywords:** General practice, Healthcare quality, Quality indicators, Chronic diseases, Quality circles

## Abstract

Guideline adherence of general practitioners (GP) regarding treatment of chronic conditions shows room for improvement. Thus, concepts have to be designed to promote quality of care. The aim of the interventional study “Improvement of Quality by Benchmarking” was to assess whether quality can be improved by self-auditing, benchmarking and quality circles in Salzburg (Austria) and South Tyrol (Italy). In this publication we present the Austrian results. Quality indicators were developed in a consensus process for eight chronic diseases based on pre-existing quality management systems. A quality score consisting of 35 indicators was calculated (0–5 points per indicator depending on fulfilment, maximum 175 points). Data were extracted from the electronic health records of participating practices in 2012, 2013 and 2014. A statistical pre-post analysis was performed using Wilcoxon signed-rank tests. A total of 20 GPs participated in the project. The mean quality score increased from 62.0 at baseline to 84.0 at the second follow-up (*p* = 0.003). Regarding the individual quality indicators, strong improvements were achieved between baseline and first follow-up, especially in process indicators concerning documentation. Between the first and second follow-up, quality remained in most cases at the same level. The validity of results is limited because of structural and technical problems. Due to the uncontrolled pre-post design we cannot exclude external influences on the results. Nevertheless, the intervention was able to improve measured quality of care. Barriers were detected that should be considered in a possible implementation of quality control programs.

## Introduction

Chronic conditions are the most important cause of illness and disability in Europe and place a high financial demand on healthcare systems [1]. Medical care of patients with chronic diseases is often challenging and requires coordinated follow-up, which is usually assured by general practitioners (GP) [2]. Nevertheless, patients with chronic conditions are not always appropriately treated and guideline adherence of GPs leaves room for improvement [3, 4]. For example, an Austrian cross-sectional study assessing 9 quality indicators in 501 patients with chronic diseases detected non-adherence to guidelines in 16.8 % of the 1224 quality indicators which could be applied, mostly due to physicians’ lack of knowledge [3]. Another study found that only 29.3 % of patients with chronic heart failure were treated with beta blockers and only 35.6 % of those patients receiving ACE-I were prescribed the recommended daily dose [4].

A key role is played by GPs in quality improvement in the healthcare system [2] as quality enhancement has been defined as an essential topic of general practice [5]. Regarding the approach strategies of quality improvement in outpatient care, several methods exist: for instance, quality circles consist of small groups of GPs who meet regularly to find solutions purposefully and autonomously for occurring problems [6]. They are an accepted instrument for GPs and have been shown to make an impact on care outcomes [7]. Peer reviews are critical (self-)reflections of medical actions in the dialogue with colleagues within the same discipline. The term peer review was originally used as a synonym for quality circles [6]. (Self-)audits consist of a systematic and critical evaluation of quality in a GP’s surgery performed by external physicians or by reflection of data by the physicians themselves, based on the available evidence or accepted consensus guidelines [8]. Another approach to improve quality is benchmarking, which is a comparative method measuring and ranking performance indicators [6]. Overall, analysis of systematic reviews shows that combined and multifaceted interventions with educational components are more effective [9].

Quality indicators (QI) have been a topic of increasing interest over the last decades enabling conclusions on current quality and on improving opportunities. They were defined by Lawrence and Olesen [10] as “a measurable element of practice performance for which there is evidence or consensus that it can be used to assess the quality, and hence change in the quality of care provided”*. *Concepts using QIs have been established in several countries. For instance, in Germany, the quality indicator system for outpatient treatment project (*Qualitätsindikatorensystem für die ambulante Versorgung*, QISA) uses a set of QIs in primary care for promoting transparency of quality [11], while in the UK the quality and outcomes framework (QOF) has been used since 2004 as a pay for performance scheme based on a large number of QIs for several chronic conditions, organization of care and patient experiences [12], generating payments according to the quality standards achieved. In Italy, quality assessments with process and intermediate outcome indicators are performed by Health Search (research unit of the Italian Society of General Practice, SIMG) providing a network of GPs the possibility to perform self-audits and to benchmark their performance with other GPs [13]. In Austrian primary care, an established systematic quality enhancement approach based on QIs does not yet exist, although quality improvement in healthcare has become an important field of political interest in recent years [14].

Against this background, the present study called „Improvement of Quality by Benchmarking“ (IQuaB) was started as a transnational quality improving initiative of GPs in Salzburg (Austria) and South Tyrol (Italy) with the aim to assess quality of care of patients with eight chronic diseases by a quality score and by itemized QIs in general practice surgeries and to assess whether the quality of healthcare can be improved by a combined intervention consisting of self-auditing, benchmarking and quality circles within 18 months. In this article we present the Austrian results. The Italian data and the comparison between the two regions will be reported elsewhere.

## Methods

The study was conducted as an uncontrolled interventional study between October 2011 and September 2014 in two study regions, two counties of the province of Salzburg, Austria and the province of South Tyrol, Italy.

### GP recruitment

As this was designed as a pre-post study, no sample size calculation was performed. We aimed at recruiting 30 GPs. All practicing family physicians were considered to be eligible if they were working alone or in group practices in primary care in the counties of Pinzgau or Pongau (Salzburg, Austria); therefore, all GPs with an address in the specified regions registered at the Salzburg Medical Chamber with or without a contract with the statutory health insurance (*n* = 114 GP practices including 133 GPs) were invited to participate by letter, email or telephone. Physicians were remunerated for participation.

### Development of QIs and quality standards

QIs were developed in a consensus process by screening several guidelines [15–18] and by adapting performance indicators used by Health Search [13] and in the QOF [19]; the following QIs were used in the IQuaB project: prevalences of diseases, recording of BMI, recording of blood pressure, registration of smoking behavior, creatinine measurement, glycosylated haemoglobin (HbA1c) measurement, HbA1c < 7.5 %, documentation of spirometry, metformin prescription, statin prescription, beta blocker prescription, ACE-I/ARB prescription, prescription of antithrombotic therapy. Several of these QIs were applied more than once because they were applicable to more than one of the eight targeted diseases. This resulted in a total number of 43 QIs (see Table 1). Several participating physicians were involved in the development process to assure the acceptance of the QIs by GPs and the feasibility of data extraction from the electronic health records (EHRs). The low number of QIs in comparison to e. g. the QOF indicators was due to the limited feasibility of extracting and measuring indicators in the EHRs used in Austria. Object of the investigation was the quality of care regarding eight common chronic diseases in general practice: type 2 diabetes mellitus (DM), hypertension (HT), coronary heart disease (CHD), cerebrovascular disease (CBVD), peripheral arterial disease (PAD), chronic heart failure (CHF), atrial fibrillation (AF) and chronic obstructive pulmonary disease (COPD). The indicators provided information about the prevalence of the diseases, documentation process, diagnostic tests and medical therapy. Time intervals for the single QIs were defined as a determined time period in which the respective documentation, diagnostic test or prescription should be performed, including an additional quarter year as the range of tolerance. As patients with chronic diseases usually visit their GP at least once a year, prevalence rates were defined (for data extraction) as the percentage of patients with a specific condition who visited the GP within the last 15 months, in relation to the total number of patients treated in the same time period by the respective GP. Since this reflects the prevalence among patients who visit the GP at least once in 15 months and not the prevalence in the general population, for data analysis the extracted prevalence rates were adjusted for the proportion of patients who do not visit the GP regularly according to results of the Austrian Health Survey 2006/2007 [20]. Time intervals for QIs regarding drug prescriptions were 3 months as the investigated chronic conditions usually require uninterrupted therapy. Exception was the prescription of antiplatelet and anticoagulant drugs, for which an 8‑month prescription interval was chosen because of low dosage intakes and large package sizes.

**Table 1:** Quality indicators and quality standards (acceptable and ideal level of performance) used in the IQuaB project

No.		Acceptable (%)	Ideal (%)
	**Diabetes mellitus type 2**		
1	Prevalence	4.5	7
2	Body mass index (recorded within last 15 months)	80.0	100.0
3	Blood pressure (recorded within last 15 months)	70.0	100.0
4	Registration of smoking behavior (smoker or non-smoker)	80.0	100.0
5	Creatinine measurement (done within last 15 months)	80.0	100.0
6	HbA1c measurement (done within last 9 months)	60.0	90.0
7	HbA1c < 7.5 % (any value within last 9 months)	70.0	90.0
8	Metformin prescription (within last 3 months) if HbA1c ≥ 7.5 % (any value within last 9 months)	70.0	90.0
	**Hypertension**		
9	Prevalence	20.0	30.0
10	Body mass index (recorded within last 15 months)	80.0	100.0
11	Blood pressure (recorded within last 15 months)	70.0	100.0
12	Registration of smoking behavior (smoker or non-smoker)	80.0	100.0
13	Creatinine measurement (done within last 15 months)	80.0	100.0
	**Coronary heart disease**		
14	Prevalence	2.0	2.5
15	Body mass index (recorded within last 15 months)	80.0	100.0
16	Blood pressure (recorded within last 15 months)	70.0	100.0
17	Registration of smoking behavior (smoker or non-smoker)	80.0	100.0
18	Statin prescription (within last 3 months)	80.0	90.0
19	Beta-blocker prescription (within last 3 months)	80.0	90.0
20	Prescription of antithrombotic therapy (within last 8 months)	80.0	90.0
	**Cerebrovascular disease**		
21	Prevalence	2.0	2.0
22	Body mass index (recorded within last 15 months)	80.0	100.0
23	Blood pressure (recorded within last 15 months)	70.0	100.0
24	Registration of smoking behavior (smoker or non-smoker)	80.0	100.0
25	Statin prescription (within last 3 months)	80.0	90.0
26	Prescription of antithrombotic therapy (within last 8 months)	80.0	90.0
	**Peripheral arterial disease**		
27	Prevalence	2.0	3.0
28	Body mass index (recorded within last 15 months)	80.0	100.0
29	Blood pressure (recorded within last 15 months)	70.0	100.0
30	Registration of smoking behavior (smoker or non-smoker)	80.0	100.0
31	Statin prescription (within last 3 months)	80.0	90.0
32	Prescription of antithrombotic therapy (within last 8 months)	80.0	90.0
	**Chronic heart failure**		
33	Prevalence	1.5	3.0
34	Body mass index (recorded within last 15 months)	80.0	100.0
35	Blood pressure (recorded within last 15 months)	70.0	100.0
36	ACE-I or ARB prescription (within last 3 months)	80.0	90.0
37	Beta-blocker prescription (within last 3 months)	80.0	90.0
	**Atrial fibrillation**		
38	Prevalence	2.0	2.0
39	Blood pressure (recorded within last 15 months)	70.0	100.0
40	Prescription of antithrombotic therapy (within last 8 months)	80.0	90.0
	**Chronic obstructive pulmonary disease**		
41	Prevalence	2.5	5.0
42	Registration of smoking behavior (smoker or non-smoker)	80.0	100.0
43	Spirometry (at least one electronic record)	70.0	100.0

*HbA1c* glycosylated hemoglobin, *ACE-I* Angiotensin-converting enzyme inhibitor, *ARB* Angiotensin receptor blocker

In the participating surgeries, five different EHRs were in use that provided restricted possibilities to extract data; therefore, only the limited number of 43 QIs could be applied. The achievement regarding a QI was defined as the percentage of patients who fulfilled the respective criterion in relation to the total number of patients with the concerning diagnosis. Exceptions were HbA1c values < 7.5 % and metformin prescriptions if HbA1c ≥ 7.5 %, which depended on the diagnosis and on another QI: the indicator HbA1c < 7.5 % was determined as the percentage of patients who had an HbA1c value below target in relation to the total number of patients with DM who had an HbA1c measurement. The QI metformin prescriptions if HbA1c ≥ 7.5 % was determined as the percentage of patients who had a metformin prescription in relation to all patients with DM who had an HbA1c measurement and a value above the target value.

For each indicator, quality standards (acceptable and ideal level of performance) were defined, describing the frequency with which the QI criterion should be attained [10]. As evidence-based quality standards do not exist, target values used in our study were determined in a consensus process based on the target values used by Health Search^1^ [13] and on the QOF payment stages [19]. All QIs and their respective target values are listed in Table 1.

### Data collection

Data extraction was performed in June–September 2012, April–June 2013 and January–April 2014. Physicians’ data were retrieved but not individual patient data (e. g. number of patients with diabetes and registration of smoking behavior per GP). The project staff conducted the extraction of data manually in the GP practices. As diagnoses are usually not coded in Austrian general practices and are recorded in the EHR as string variables, GPs were asked to standardize the terminology of diagnoses (e. g. DM2 instead of type 2 diabetes mellitus) to simplify the search. Also medications are not usually recorded using ATC codes and were therefore searched via brand names as strings. It was not feasible to retrieve medication plans for long-term drug treatment from the EHR in all surgeries because they were not consistently entered by GPs. Hence, in many cases, single prescriptions within the respective predetermined time-range had to be extracted. Laboratory values, smoking behavior and blood pressure also had to be searched using strings in most cases because the data and measurements were not recorded in standardized data fields.

The five EHR systems required different search strategies and enabled various degrees of data extraction. In EHR 1, entering multiple search terms was not possible and each medication had to be searched for separately. Word root searches were also used, e. g. “simva” and “statin”. All searching results were summed up manually; however, compound terms (e. g. simvastatin) were counted twice and were therefore searched additionally and then subtracted. For the most used software (EHR 2), an additional data filtering module was acquired for the study period, which allowed several search terms to be used and to combine them by an operator and counting every patient only once; however, the number of search terms was limited by a maximum number of characters. Diagnoses could also be searched as permanent diagnoses. Search profiles could be saved and used for the next data extraction with adaptation of the search interval. In EHR 3, extraction of prescriptions was only possible if a diagnosis was recorded within the last 3 months. Otherwise, the patient was not counted. The company producing EHR 4 provided searching profiles for Microsoft® Access 2010, where data had to be transferred and anonymized allowing extraction without any restraints. The EHR 5 only allowed the extraction of the prevalences; therefore, all other QIs resulted as missing values in the respective GPs. The filter allowed only data within a specific year to be extracted and not within the last 15 months, as defined in our study; however, we assumed that this extraction process did not overlook patients, as patients with chronic illnesses usually visit their GP at least once a year.

Due to the heterogeneous and limited technical opportunities, standardization of the data extraction was not possible. To achieve a minimum of standardization, search terms for diagnoses and medications were listed for data extraction. Three GPs using EHR 1 and 3 were excluded from data analysis because extraction was not possible according to the definition of QIs.

### Ethics

The ethics committee of the province of Salzburg gave an ethics waiver because no individual patient data were collected or processed.

### Intervention

After each data extraction all GPs received graphic and written information about their fulfilment of QIs in percentages (self-audit) and anonymously the results of their colleagues (benchmarking). Furthermore, the medians of the region and the quality standards previously defined (acceptable and ideal level of performance for all QIs) were provided. The report was sent per postal delivery.

Regional quality circles of the participating GPs with support of the project team were established after the first data collection to discuss results, differences between regions and physicians and to elaborate possibilities for improvement in chronic care. Quality circles were conducted on average once or twice a year, attendance was optional. Two transnational quality circles (September 2013 and May 2014) were additionally organized for all GPs from both regions to enhance networking between the countries.

### Data analysis

Benchmarking analysis was conducted manually using Microsoft® Excel® 2010.

Calculation of the quality score: the quality score was developed by the project team as follows and calculated using IBM® SPSS® statistics version 20.0. We considered all quality indicators except prevalence rates, as we could not find representative Austrian comparative data for all eight diseases targeted by IQuaB and the prevalence could be biased by overdiagnosis or underdiagnosis (in contrast to the other QIs that are generally better the higher they are). Therefore, 35 QIs were included in the calculation of the quality score. We assigned between zero and five points per QI. Point assignment for each indicator was based on the median at baseline: the median minus 10 % was the minimum to achieve one point. If the median fell below 20 %, 10 % was the minimum to achieve one point (e. g. recording of smoking behavior in diabetes). No point was awarded if the calculated minimum target value was not reached. For each additional 5 % that were reached, one further point was added. For simplification, percentages were rounded. For each GP, one individual quality score was calculated by summation of the points of the single QIs. Quality scores could not be calculated for the three GPs using EHR 5, from which only prevalence rates could be extracted.

Missing data and exclusion of data: if prevalence rates were 0, we excluded all other QIs depending on the prevalence. As the data extraction was performed manually, some errors in the data extraction process occurred. In the cases where the mistakes could not be corrected during the data extraction process or afterwards, values that could not be valid (e. g. percentages that exceeded 100 %) were excluded.

Statistical analysis was carried out using the software package IBM® SPSS® statistics version 20.0. Units of analyses were GPs. To check for normal distribution, the Kolmogorov Smirnov test was used. As data were skewed, we performed Wilcoxon signed-rank tests for longitudinal analysis. Significance level was set at 5 % (*p* < 0.05). Tests were carried out with the quality score and the single QIs.

## Results

### Participating physicians and EHR

**Fig. 1:**
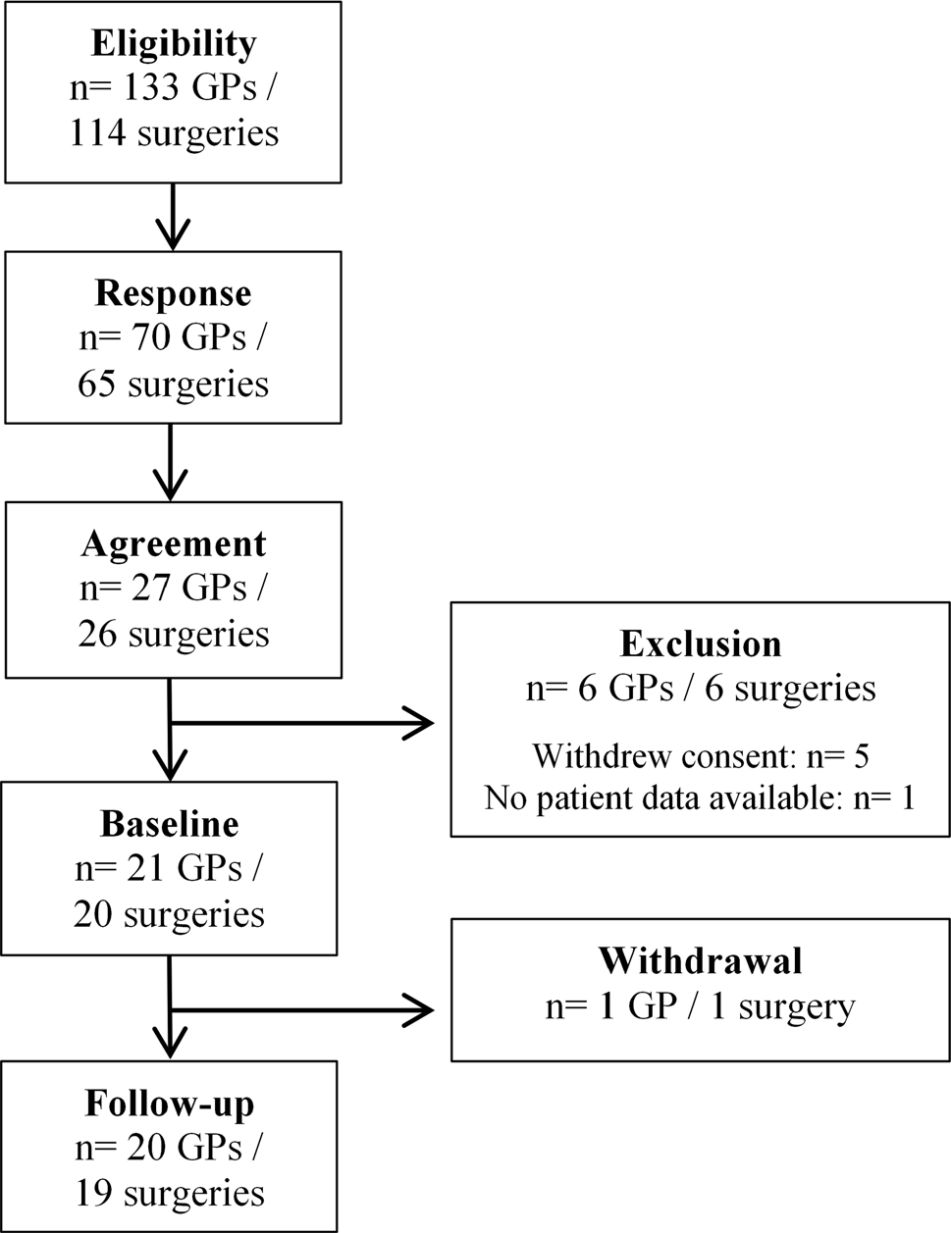
Flow chart of GP recruitment and participation

Of the 114 surgeries (133 GPs) invited to participate in Salzburg, 26 surgeries (27 GPs) signed informed consent for the study (participation rate of surgeries 22.8 %), 19 surgeries (20 GPs) finished the study (Fig. 1). 30.0 % of the participating GPs were female, 50.0 % of the participating GPs surgeries were located in the province Pongau and 50.0 % were located in the province Pinzgau. The mean age of participating physicians was 52.9 years. All but one of the participating GPs worked in single-handed practices. The participating GPs were of a similar sex and regional distribution as all invited GPs (26.3 % female, 51.1 % Pongau). We could not obtain any further data from non-participating GPs so that a more detailed comparison was not possible.

The distribution of the EHRs used by the GPs is listed in Table 2.

**Table 2:** The use of electronic health records among participating GPs (status as of 2012)

Electronic health records	Number of surgeries (GPs)
EHR 1	1 (1)
EHR 2	11 (12)
EHR 3	2 (2)
EHR 4	3 (3)
EHR 5	3 (3)

### Quality score

**Fig. 2:**
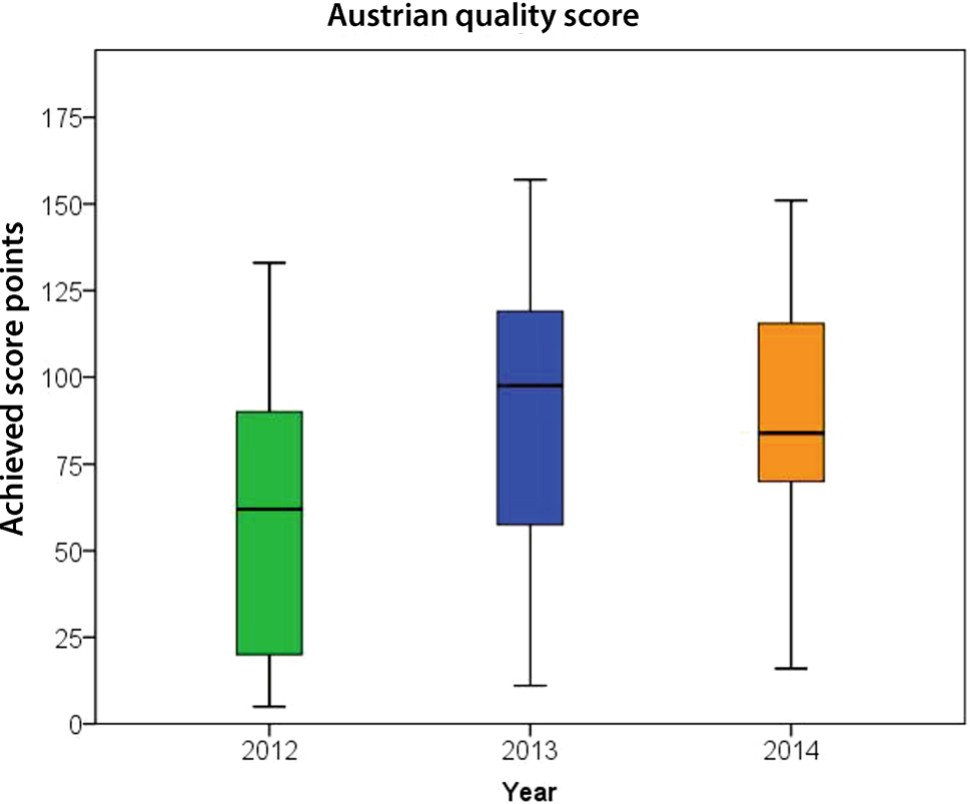
Longitudinal analysis of the quality score. Median quality score 2012: 62.0 points (Q_1_–Q_3_: 19.5–99.5), median quality score 2013: 97.5 points (Q_1_–Q_3_: 55.3–119.0), median quality score 2014: 84.0 points (Q_1_–Q_3_: 69.5–119.8)

Quality score point assignments and cut-offs for the considered 35 QIs are shown in Table 3. In total, 175 points were achievable (up to 5 points for each QI). The median quality score (Fig. 2) increased significantly from baseline to the first follow-up (*p* = 0.002) and decreased not significantly between first and second follow-up (*p* = 0.535); however, improvement from baseline to second follow-up remained significant (*p* = 0.003).

**Table 3:** Spreadsheet for the calculation of the quality score (point assignment based on the median value at baseline for each quality indicator)

	Median (%)	0 point	1 point	2 points	3 points	4 points	5 points
**Diabetes mellitus type 2**							
Body mass index (recorded within last 15 months)	18.4	<10	10.0–14.9	15.0–19.9	20.0–24.9	25.0–29.9	≥30
Blood pressure (recorded within last 15 months)	56.9	<45	45.0–49.9	50.0–54.9	55.0–59.9	60.0–64.9	≥65
Registration of smoking behavior (smoker or non-smoker)	4.1	<10	10.0–14.9	15.0–19.9	20.0–24.9	25.0–29.9	≥30
Creatinine measurement (done within last 15 months)	46.8	<35	35.0–39.9	40.0–44.9	45.0–49.9	50.0–54.9	≥55
HbA1c measurement (done within last 9 months)	55.4	<45	45.0–49.9	50.0–54.9	55.0–59.9	60.0–64.9	≥65
HbA1c < 7.5 % (any value within last 9 months)	74.0	<65	65.0–69.9	70.0–74.9	75.0–79.9	80.0–84.9	≥85
Metformin prescription (within last 3 months) if HbA1c ≥ 7.5 % (any value within last 9 months)	53.8	<45	45.0–49.9	50.0–54.9	55.0–59.9	60.0–64.9	≥65
**Hypertension**							
Body mass index (recorded within last 15 months)	16.3	<10	10.0–14.9	15.0–19.9	20.0–24.9	25.0–29.9	≥30
Blood pressure (recorded within last 15 months)	50.6	<40	40.0–44.9	45.0–49.9	50.0–54.9	55.0–59.9	≥60
Registration of smoking behavior (smoker or non-smoker)	5.8	<10	10.0–14.9	15.0–19.9	20.0–24.9	25.0–29.9	≥30
Creatinine measurement (done within last 15 months)	41.0	<30	30.0–34.9	35.0–39.9	40.0–44.9	45.0–49.9	≥50
**Coronary heart disease**							
Body mass index (recorded within last 15 months)	24.4	<15	15.0–19.9	20.0–24.9	25.0–29.9	30.0–34.9	≥35
Blood pressure (recorded within last 15 months)	53.5	<45	45.0–49.9	50.0–54.9	55.0–59.9	60.0–64.9	≥65
Registration of smoking behavior (smoker or non-smoker)	4.5	<10	10.0–14.9	15.0–19.9	20.0–24.9	25.0–29.9	≥30
Statin prescription (within last 3 months)	36.9	<25	25.0–29.9	30.0–34.9	35.0–39.9	40.0–44.9	≥45
Beta-blocker prescription (within last 3 months)	30.5	<20	20.0–24.9	25.0–29.9	30.0–34.9	35.0–39.9	≥40
Prescription of antithrombotic therapy (within last 8 months)	69.7	<60	60.0–64.9	65.0–69.9	70.0–74.9	75.0–79.9	≥80
**Cerebrovascular disease**							
Body mass index (recorded within last 15 months)	24.2	<15	15.0–19.9	20.0–24.9	25.0–29.9	30.0–34.9	≥35
Blood pressure (recorded within last 15 months)	52.3	<40	40.0–44.9	45.0–49.9	50.0–54.9	55.0–59.9	≥60
Registration of smoking behavior (smoker or non-smoker)	5.6	<10	10.0–14.9	15.0–19.9	20.0–24.9	25.0–29.9	≥30
Statin prescription (within last 3 months)	29.6	<20	20.0–24.9	25.0–29.9	30.0–34.9	35.0–39.9	≥40
Prescription of antithrombotic therapy (within last 8 months)	65.0	<55	55.0–59.9	60.0–64.9	65.0–69.9	70.0–74.9	≥75
**Peripheral arterial disease**							
Body mass index (recorded within last 15 months)	25.0	<15	15.0–19.9	20.0–24.9	25.0–29.9	30.0–34.9	≥35
Blood pressure (recorded within last 15 months)	47.2	<35	35.0–39.9	40.0–44.9	45.0–49.9	50.0–54.9	≥55
Registration of smoking behavior (smoker or non-smoker)	0.0	<10	10.0–14.9	15.0–19.9	20.0–24.9	25.0–29.9	≥30
Statin prescription (within last 3 months)	34.0	<25	25.0–29.9	30.0–34.9	35.0–39.9	40.0–44.9	≥45
Prescription of antithrombotic therapy (within last 8 months)	74.2	<65	65.0–69.9	70.0–74.9	75.0–79.9	80.0–84.9	≥85
**Chronic heart failure**							
Body mass index (recorded within last 15 months)	20.0	<10	10.0–14.9	15.0–19.9	20.0–24.9	25.0–29.9	≥30
Blood pressure (recorded within last 15 months)	60.0	<50	50.0–54.9	55.0–59.9	60.0–64.9	65.0–69.9	≥70
ACE-I or ARB prescription (within last 3 months)	43.8	<35	35.0–39.9	40.0–44.9	45.0–49.9	50.0–54.9	≥55
Beta-blocker prescription (within last 3 months)	29.4	<20	20.0–24.9	25.0–29.9	30.0–34.9	35.0–39.9	≥40
**Atrial fibrillation**							
Blood pressure (recorded within last 15 months)	57.5	<45	45.0–49.9	50.0–54.9	55.0–59.9	60.0–64.9	≥65
Prescription of antithrombotic therapy (within last 8 months)	67.8	<60	60.0–64.9	65.0–69.9	70.0–74.9	75.0–79.9	≥80
**Chronic obstructive pulmonary disease**							
Registration of smoking behavior (smoker or non-smoker)	13.1	<10	10.0–14.9	15.0–19.9	20.0–24.9	25.0–29.9	≥30
Spirometry (at least one electronic record)	11.7	<10	10.0–14.9	15.0–19.9	20.0–24.9	25.0–29.9	≥30

*HbA1c* glycosylated hemoglobin, *ACE-I* Angiotensin-converting enzyme inhibitor, *ARB* Angiotensin receptor blocker

### Explorative analysis of individual QIs

A significant increase of prevalence was observed in DM from the first to the second follow-up and over the study period in HT, AF and COPD. The BMI recordings increased enormously but not significantly from baseline to first follow-up in HT and CHD and decreased significantly from first to second follow-up. In PAD and CHF, the percentage of patients with BMI recordings increased significantly from baseline to first follow-up and declined afterwards not significantly. Blood pressure measurements increased in DM, CHD and PAD significantly from baseline to first follow-up and additionally from baseline to second follow-up in CHD and PAD. Registration of smoking behavior increased significantly from baseline to first and second follow-up in all concerning diseases but HT. The percentage of creatinine measurement did not change significantly. The HbA1c measurements, HbA1c values below 7.5 % and metformin prescriptions if HbA1c ≥ 7.5 % improved slightly but not significantly over the study period. Spirometry in patients with COPD increased significantly from baseline to second follow-up, although there was a significant decline from first to second follow-up.

The ACE-I or ARB prescriptions as well as prescriptions of antithrombotic agents did not alter in any of the diseases concerned. Beta blocker prescriptions increased significantly in CHD patients but not in CHF patients. Statin prescriptions in CHD improved significantly over the study period but did not change in CBVD and in PAD. The results for all QIs are shown in Table 4.

**Table 4:** Results of the longitudinal analysis of all quality indicators (median, first and third quartile in percentages and *p*-values) (*italic numbers* significant increase, **bold numbers **significant decrease)

Quality indicator	2012			2013			2014			*p*-value		
	Median	Q_1_	Q_3_	Median	Q_1_	Q_3_	Median	Q_1_	Q_3_	2012–2013	2013–2014	2012–2014
**Diabetes mellitus type 2**												
Prevalence	3.9	2.5	5.1	4.3	2.6	5.4	4.8	3.4	5.7	0.469	*0.018*	0.117
Body mass index (recorded within last 15 months)	18.4	6.5	69.1	24.0	9.7	77.2	22.7	3.2	59.5	0.075	0.110	0.534
Blood pressure (recorded within last 15 months)	56.9	33.0	76.6	62.5	32.3	80.8	68.9	31.0	87.0	*0.008*	0.605	0.125
Registration of smoking behavior (smoker or non-smoker)	4.1	0.0	9.7	12.9	8.0	22.0	20.0	9.1	22.7	*0.011*	0.799	*0.007*
Creatinine measurement (done within last 15 months)	46.8	31.9	72.5	55.9	37.2	78.3	60.8	36.2	71.8	0.255	0.501	0.717
HbA1c measurement (done within last 9 months)	55.4	39.5	77.4	54.3	40.5	78.8	62.9	50.3	77.9	0.826	0.433	0.331
HbA1c < 7.5 % (any value within last 9 months)	74.0	31.1	93.5	81.0	54.8	89.1	83.6	60.9	89.6	0.768	0.173	0.515
Metformin prescription (within last 3 months) if HbA1c ≥ 7.5 % (any value within last 9 months)	53.9	19.8	82.1	78.6	45.1	87.3	63.2	38.1	85.2	1.000	0.575	0.859
**Hypertension**												
Prevalence	14.3	9.6	16.3	15.4	12.8	19.3	15.9	13.6	20.0	*0.039*	0.267	*0* *.003*
Body mass index (recorded within last 15 months)	16.3	6.2	53.1	42.7	12.3	60.1	22.2	5.0	49.5	0.182	**0.006**	0.155
Blood pressure (recorded within last 15 months)	50.6	39.1	75.3	67.9	37.9	85.8	71.5	34.3	86.6	0.583	0.691	0.754
Registration of smoking behavior (smoker or non-smoker)	5.8	0.8	11.2	18.0	11.5	23.1	13.9	7.0	24.9	0.110	0.169	0.114
Creatinine measurement (done within last 15 months)	41.0	18.5	52.0	41.6	25.8	57.9	38.1	15.6	50.3	0.469	0.163	0.148
**Coronary heart disease**												
Prevalence	2.9	1.3	4.9	2.9	1.8	4.5	2.9	1.8	4.5	0.841	0.717	0.936
Body mass index (recorded within last 15 months)	24.4	6.5	51.7	44.9	6.7	61.6	20.2	3.3	53.7	0.062	**0.034**	0.937
Blood pressure (recorded within last 15 months)	53.5	44.8	77.0	59.9	43.9	86.4	65.9	47.5	86.8	*0.041*	0.173	*0.008*
Registration of smoking behavior (smoker or non-smoker)	4.6	0.0	5.7	20.0	10.2	28.7	23.6	12.9	31.8	*0.011*	0.959	*0.005*
Statin prescription (within last 3 months)	36.9	27.8	48.7	43.8	30.9	57.4	43.5	29.5	58.4	0.096	0.551	*0.035*
Beta-blocker prescription (within last 3 months)	30.5	22.8	40.1	41.5	32.7	55.7	42.8	33.3	64.7	*0.005*	0.221	*0.001*
Prescription of antithrombotic therapy (within last 8 months)	69.7	57.3	75.9	68.7	56.3	77.6	66.4	58.9	80.3	0.177	0.975	0.245
**Cerebrovascular disease**												
Prevalence	1.3	0.9	2.0	1.5	1.0	1.9	1.4	1.0	2.0	0.295	0.520	0.469
Body mass index (recorded within last 15 months)	24.2	2.3	49.9	45.4	13.1	54.0	23.6	10.4	50.9	0.136	0.158	0.638
Blood pressure (recorded within last 15 months)	52.3	39.0	73.3	66.0	31.7	81.5	54.0	25.0	71.4	0.272	0.198	0.422
Registration of smoking behavior (smoker or non-smoker)	5.6	0.0	11.1	24.9	11.1	43.6	13.2	7.6	31.3	*0.008*	0.074	*0.028*
Statin prescription (within last 3 months)	29.6	17.3	36.5	28.7	22.0	40.3	28.6	21.4	40.1	0.875	0.875	0.552
Prescription of antithrombotic therapy (within last 8 months)	65.0	56.0	71.1	66.7	57.9	75.6	64.6	50.0	70.0	0.552	0.331	0.433
**Peripheral arterial disease**												
Prevalence	0.6	0.3	0.9	0.5	0.3	0.9	0.6	0.3	0.9	0.647	0.831	0.913
Body mass index (recorded within last 15 months)	25.0	7.7	52.3	38.7	8.5	56.8	20.0	0.0	58.0	*0.047*	0.328	0.248
Blood pressure (recorded within last 15 months)	47.2	32.5	77.1	67.4	34.0	89.7	76.0	43.2	84.6	*0.026*	1.000	*0.033*
Registration of smoking behavior (smoker or non-smoker)	0.0	0.0	20.0	41.9	19.4	58.4	41.7	18.2	60.0	*0.008*	0.093	*0.013*
Statin prescription (within last 3 months)	34.0	19.0	50.0	38.8	28.7	55.4	39.6	35.2	52.8	0.084	0.875	0.117
Prescription of antithrombotic therapy (within last 8 months)	74.2	63.5	80.3	82.6	71.3	93.0	77.4	71.5	88.4	0.050	0.158	0.507
**Chronic heart failure**												
Prevalence	0.6	0.2	0.8	0.5	0.3	1.2	0.5	0.3	1.0	0.198	0.198	0.841
Body mass index (recorded within last 15 months)	20.0	0.0	57.6	33.3	17.7	73.1	31.3	8.9	65.7	*0.011*	0.646	0.374
Blood pressure (recorded within last 15 months)	60.0	26.9	86.4	77.6	55.9	95.7	82.9	55.7	95.9	0.182	0.917	0.155
ACE-I or ARB prescription (within last 3 months)	43.8	18.7	60.5	50.0	43.0	64.1	51.1	32.7	59.1	0.374	0.583	0.445
Beta-blocker prescription (within last 3 months)	29.4	9.8	48.5	42.3	33.3	60.0	47.2	30.8	60.2	0.093	0.859	0.074
**Atrial fibrillation**												
Prevalence	1.6	1.0	2.8	2.3	1.7	3.0	2.6	1.3	3.1	0.064	0.227	*0.027*
Blood pressure (recorded within last 15 months)	57.5	38.7	76.2	63.9	53.2	83.8	64.1	42.3	75.6	0.470	0.551	0.551
Prescription of antithrombotic therapy (within last 8 months)	67.8	58.5	76.3	75.6	70.7	79.6	75.0	64.8	79.1	0.074	0.683	0.140
**Chronic obstructive pulmonary disease**												
Prevalence	1.8	1.3	3.3	1.9	1.5	3.7	2.1	1.5	4.1	*0.044*	0.126	*0.020*
Registration of smoking behavior (smoker or non-smoker)	13.1	2.7	39.6	56.0	38.6	69.0	52.4	39.1	58.3	*0.011*	0.594	*0.007*
Spirometry (at least one electronic record)	11.7	3.7	17.7	45.1	9.5	82.7	42.2	11.6	83.2	*0.018*	**0.036**	*0.018*

*HbA1c* glycosylated hemoglobin, *ACE-I* Angiotensin-converting enzyme inhibitor, *ARB* Angiotensin receptor blocker

### Achievement of quality standards

QIs at baseline were below the set quality standards (acceptable level of performance) except of the median percentage of HbA1c < 7.5 % in DM that amounted between 70 and 90 % and remained in this range. Prescription of antithrombotic therapy in PAD patients and metformin prescriptions achieved the quality standards in the first follow-up, but fell below again at the second follow-up. Blood pressure measurements within the last 15 months in patients with CHF were rising throughout the study period and achieved the quality standards at the first and second follow-up. Measurements of blood pressure in HT and PAD as well as DM prevalence achieved the determined quality standard at the second follow-up. The percentage of HbA1c measurements were close below the set level of acceptable performance and achieved it at the second follow-up.

## Discussion

### Summary and interpretation of findings

Baseline performance as measured by the quality score was low. Improvement of the quality score was remarkable in the period between baseline and first follow-up. Taking a closer look to the individual QIs, we can observe that especially documentation indicators increased (e. g. registration of smoking behavior), while prescriptions only changed to some extent. The only measured intermediate outcome parameter (HbA1c < 7.5 %) increased slightly but not significantly; however, this indicator was already at a relatively high level at baseline. While in the period between baseline and first follow-up strong improvements were reached, in the period from first to second follow-up improvements slowed down or quality of care remained at the same level in many cases. Some QIs were even regressive between first and second follow-up; however, most of the significant improvements achieved up until the first follow-up remained significant between baseline and the second follow-up. Wide spans between first and third quartile indicated large differences among GPs.

The low grade of QI fulfilment in our sample may be related to different possible scenarios: firstly, clinical actions were actually not performed, secondly, clinical actions were performed but were not documented in the EHR and thirdly, clinical actions were performed and documented but it was not possible to extract the information from the EHR. We assume that all three explanatory models played a role in our sample but a case-based assignment was not possible. Even medications that are usually electronically prescribed by a GP may be prescribed by other medical specialists intermittently or prescribed by hand during home visits. In this case, the medication does not appear in the EHR despite regular prescription. Another example is that we assume that many GPs particularly in rural areas know whether their patients smoke and are aware of the risk factor but often do not systematically record smoking behavior and thus have “low performance” in the benchmarking. Furthermore, the lack of possibility for exception reporting as it is possible in the UK (e.g. deliberate exclusion of patients as in the case of contraindications) might have contributed to the low prescription rates. Exclusion of patients leads to a lower denominator in the calculation of the respective QI and thus, to a higher percentage of achievement. The fact that we did not incentivise the achievement of quality standards potentially strengthens the low grade of fulfilment. The remarkable improvements between baseline and first follow-up could be due to increased awareness and documentation on the side of GPs but could also reflect a true improvement of performance.

We considered possible factors leading to lower QI fulfilment, which do not depend on the performance of the GPs (e. g. known percentages of contraindications or patient non-compliance), at the beginning of the study by setting the levels of the target values below 100 % according to the standards used by Health Search [13]. Nevertheless, the target levels were only occasionally achieved. Hence, besides performance and documentation factors, also the levels of the quality standards might be a point of discussion.

### Results in the context of similar studies and quality programs

The IQuaB results can be only partially compared to other Austrian data as there is a lack of representative epidemiological data and QIs are not used regularly in Austrian primary care. Epidemiological surveys of type 2 DM estimate its prevalence to be approximately 8.0–9.0 % including 2.0–3.0 % patients as yet undetected [21]. In our sample the prevalence rates were between 3.9 and 4.8 %. As our study was limited to two rural regions of Salzburg, the difference is possibly related to geographic factors (or to the abovementioned explanatory models). A study conducted in 23 GP surgeries and 1 practice of internal medicine found higher rates of HbA1c measurements (74 % within last 6 months) and serum creatinine measurements (84 % within last 12 months). The number of patients with good HbA1c control was similar; however, the sample was limited to geriatric patients [22]. The prevalence of COPD was 7.5 % in general practices in Salzburg (Austria) in a population aged 40 years or older. Only one out of five patients suffering from COPD reported a prior COPD diagnosis by the physician. This fact was interpreted as underdiagnosing [23]. In our study, the prevalence of COPD was between 1.8 and 2.1 % in the general population, which could be interpreted that COPD is also underdiagnosed in our sample. Among the subjects from another study in Salzburg, who reported a prior physician diagnosis of COPD, 68 % reported a lung function test at some time in the past [24], whereas in our sample spirometry was documented only in 12–45 % of affected patients. Some 85 % of patients in the heart failure registry in Austria (*n* = 1648 patients in ambulances of hospitals and of specialists practices) received ACE-I/ARB and 79 % received beta blockers [25]. Our results were much lower (ACE-I/ARB 44–51 % and beta blockers 29–47 %).

In England, comprehensive quality initiatives started several years before we conducted our study in Austria and Italy. After introduction of the QOF quality improvement in UK was remarkable 1 year later but slowed down after the first year and stalled when targets were reached [26]. Similarly, our results showed a high increase between baseline and first follow-up but did not improve further between first and second follow-up.

### Limitations

Precondition for measurement of QIs is the documentation and registration of diagnoses and services in the EHR. Structural challenges made the extraction of valid and reliable data difficult [27]. In Austrian primary care, most parameters are not entered in a standardized way and functions of filtering systems in EHRs are limited. Thus, values have to be interpreted cautiously. Furthermore, the technical restrictions allowed applying only a limited number of QIs. As there is no list system in Austria, the calculation of prevalence in the general population is only an extrapolation and QIs using prevalence are not reliable.

The quality score was developed with available data for comparison in retrospect based on the median value of each QI. Scoring was not based on the predefined quality standards because the percentages of QI fulfilment achieved were in most cases considerably below the standards and therefore, the quality scores would have resulted in very small numbers and depiction of changes would have been limited due to the floor effect.

Another limitation is that we mainly assessed process indicators and only one surrogate outcome and the quality score focuses on indicators of documentation quality. Although it seems to adequately reflect the progress of performance in our data at the level of GPs, the quality score does not mirror quality in its wide spectrum. The QIs in general depict only a part of real life medical care and they are not able to comprise the complexity of healthcare, especially within the multidimensional approach of family medicine [28].

We could not provide evidence on patient-relevant outcomes, such as preventing amputation, hospitalization or mortality. Up to now it remains unclear whether and how QI-driven programs influence patient-relevant outcomes. In the UK high performance in the QOF was shown to be slightly to moderately associated with good patient experience [29, 30] but a rigorous proof of a relevant effect on outcome is not yet available. A small association between QOF scores and emergency admissions [31, 32] and mortality [31] was found but results were inconsistent [31, 32]. Another study did not find a relationship between incentive payments and lives saved or quality adjusted life years [33].

Our results are not representative for Austria because GPs were recruited in specific regions, the sample is small and may over-represent highly motivated GPs. Generalizability of our results is therefore limited. Multiple testing of QIs has led to a higher chance of significant results; therefore, the interpretation of the improvement of single QIs has to be confirmed in further studies. As our study design did not contain a control group, we cannot exclude other effects on the changes beside the intervention.

### Barriers experienced

We perceived several barriers during the attempt to implement our quality improvement program. Structured quality management is relatively new in ambulatory care in Austria and viewed with scepticism. The first barrier, therefore, was general participation. Low participation of GPs is probably the result of a lack of awareness, of scepticism regarding the potential achievements of structured quality work as well as of a high workload. The GPs’ workload was also one of the most frequently given reasons for not attending the quality circles. The second barrier is that Austrian EHRs are not intended to measure QIs. Documentation in EHRs in Austrian general practices is unstructured and diagnoses and reasons for consultation are not coded. Several EHRs with heterogeneous software functions are in use. Missing software functions, e. g. for standardized entry of smoking behavior, blood pressure or laboratory values are prevalent which lead to individual solutions in each surgery. In this situation, accurate data administration is time-consuming and does not appear to be beneficial for daily work at first sight; therefore, in IQuaB, data extraction had to be performed manually by the project staff, which was technically challenging and time-consuming and thus it may have been error-prone with no possibility to estimate the magnitude of this error. All these facts impeded a standardized, valid data extraction [27].

### Implications for the future

Awareness for structured quality management (e.g. measurement of QIs, benchmarking and quality circles) should be increased in companies developing EHRs, stakeholders as well as in providers. A quality strategy for general practice in Austria would be advantageous to increase awareness and to take actions for quality improvement. Although QIs show the mentioned limitations, they are important instruments for assessing and quantifying quality [6]; improved software functions would allow more dimensions of quality to be analyzed, including (intermediate) outcome parameters. Information technology (IT) solutions should provide an intuitive user interface and relieve GPs’ daily work by facilitating standardized documentation and coding (e. g. of diagnoses, medication, laboratory values, anthropometrical data and smoking habits), by providing information (e. g. up to date guidelines, diagrams of medical parameters and benchmarking with colleagues), by supporting decisions using reminders and alerts (e. g. medication interactions) and by importing data from relevant providers, such as laboratory results. A reasonable documentation of medical data should be assured and ideally analysis of epidemiological data (e. g. prevalence, age, sex, diagnostic and therapy data) should be facilitated. The possibility of a central, standardized and valid data acquisition and processing is an essential precondition for quality promotion based on medical data. A list system in Austria could enable epidemiological studies and show real prevalence data.

Trainings for GPs in (software-assisted) quality management could be helpful to increase awareness. The mentioned barriers should be taken into account in future quality programs and IT solutions.

## Conclusion

This study was the first to implement self-auditing, benchmarking and quality circles targeted at QIs among GPs in Austria and to assess the feasibility of such a program. Low baseline performance emphasizes the importance of quality improvement initiatives in Austria. We observed strong improvements in the first study period that underline the effectiveness of our intervention and show that improvements are achievable within short periods. Although there are several limitations, our work can form the basis to develop and refine quality promotion initiatives. We identified weak structures for implementing a QI-driven program so that IT solutions and the use of coding systems are required to realize a national program based on QIs. In this case influencing factors (e. g. patient sex and age) could be taken into consideration and used additionally for risk adjustment in the benchmarking. We recommend periodically modifying the QIs because the strongest improvements are achieved within the first 9 months after implementation. Further international, long-term prospective studies are required to set evidence-based quality standards and to confirm the association between improvement of QIs and patient-relevant outcomes, such as preventing hospitalization and reducing mortality.

## Abbreviations

ACE-I: Angiotensin-converting enzyme inhibitor
AF: Atrial fibrillation
ARB: Angiotensin receptor blocker
ATC: Anatomical therapeutic chemical classification system
BMI: Body mass index
CBVD: Cerebrovascular disease
CHD: Coronary heart disease
CHF: Chronic heart failure
COPD: Chronic obstructive pulmonary disease
DM: Diabetes mellitus type 2
DMP: Disease management program
EHR(s): Electronic health record(s)
GP(s): General practitioner(s)
HT: Hypertension
NHS: National Health Service
PAD: Peripheral arterial occlusive disease
QI(s): Quality indicator(s)
QOF: Quality and outcomes framework
SIMG: Societá Italiana Medicina Generale (Italian Society of General Medical Practice)

## Footnotes

1 Target values from Health Search (ideal and acceptable level of performance) are based on the actual levels of performance of GPs measured by Health Search and on an estimated maximum of percentage achievable for each indicator, considering specific problems not depending on the GP’s purpose or knowledge (e. g. known % of contraindications or intolerances of drugs, probability of patients’ refusal). This approach offers to GPs the possibility to identify strengths and weaknesses in their care, although the target levels are disputable for being partially arbitrary and they may not make claim for completeness [13].
